# Collagen IV immunohistochemistry and autoimmune bullous diseases: defining the location of the subepidermal bullae^☆^

**DOI:** 10.1016/j.abd.2024.04.014

**Published:** 2025-01-10

**Authors:** Lula María Nieto-Benito, Verónica Parra-Blanco, Ricardo María Suárez-Fernández

**Affiliations:** a Dermatology Department, Clinica Universidad de Navarra, Madrid, Spain; b Pathology Department, Dermatopathology, Hospital General Universitario Gregorio Marañón, Madrid, Spain; c Dermatology Department, Dermatopathology, Hospital General Universitario Gregorio Marañón, Madrid, Spain

Dear Editor,

Bullous pemphigoid (BP) is the most common autoimmune blistering disease (ABD).^1^ Compatible clinical, histopathologic, and immunological criteria are necessary to differentiate among ABD.^1^, ^2^ Particularly, among subepidermal types, the level of cleavage of the blister and the exact target of the pathogenic autoantibodies (pointed out by deposition of the immunoreactions) are necessary to make a correct diagnosis.^1^

BP and epidermolysis bullosa acquisita (EBA) may resemble clinical and histopathologically. Further studies, such as Indirect Immunofluorescence (IIF) and/or Direct Immunofluorescence (DIF) in a patient’s salt-split skin, are needed to distinguish between these two entities.^1^, ^2^ However, techniques require fresh frozen tissue and sophisticated laboratory equipment.^3^

Immunohistochemistry (IHC) has also been used to study the location of basal membrane components and the presence of immunoreactions in ABD, particularly in BP.^3^, ^4^, ^5^ Collagen IV is a main component of the dermal-epidermal junction, mostly found at the *lamina densa*.^5^ Performing collagen IV IHC on formalin-fixed paraffin-embedded skin biopsies offers an inexpensive means to distinguish among subepidermal ABD.^3^, ^4^, ^5^, ^6^

We aimed to evaluate the usefulness of collagen IV IHC in BP cases attended at our center. Therefore, a retrospective, observational study that included all BP patients diagnosed between January 2000 and June 2020 was conducted.

The diagnosis of BP cases had been based on the combination of criteria encompassing the presence of compatible clinical features, typical histopathological findings, and the presence of a compatible DIF pattern (C3, IgG, IgM, and/or IgA linear deposits along the dermo-epidermal junction) and/or positive IIF/Enzyme-Linked Immunosorbent Assay (ELISA) (detection of circulating IgG anti-epidermal basement membrane antibodies by IIF microscopy studies using NaCl-separated normal human skin and/or detection of anti-BP180 and/or anti-BP230 IgG antibodies by ELISA [MESACUP anti-skin profile test]).

All cases with a confirmed diagnosis were included if, at least, three out of the four criteria were present and compatible: clinical, histopathological, serological, including IIF and/or ELISA and DIF. Skin biopsy with H&E stain and DIF was performed in all patients included in the study and results of at least one immunological study (IIF and/or ELISA) had to be present.

To assess the level of the blister and to differentiate BP from other subepidermal bullous diseases, type IV collagen immunohistochemical staining was performed in formalin-fixed paraffin-embedded tissue sections (Diagnostic Biosystems Mob229/PDM276, Mouse AntiHuman Collagen IV, purified, polyclonal antibody, IgG1, dilution 1/10-1/40). Staining at the dermal portion of the blister (floor) was considered to be compatible with BP.

From a total of 257 BP cases collected, 194 patients, in which collagen IV IHC was performed, were included (Table 1); in the remaining 63, this technique was not yet available at our center.

**Table 1:** Epidemiological and clinicopathological characteristics of included collagen IV IHC BP cases.

	Number of pacientes, n (%)
	n = 194
**Male**	116 (59.8%)
**Age** (mean ± SD) (years)	75.89 (±9.57 DS)
**Neurological comorbidities**	77 (39.7)
**Neoplastic comorbidities**	36 (18.6)
**Diagnosis of DM2**	80 (41.2)
**Clinical presentation**	
Generalized	55 (28.4)
Trunk and extremities	102 (52.5)
Trunk	10 (5.2)
Extremities	26 (13.4)
Other	1 (0.5)
**Scalp involvement**	16 (9.8)
**Mucosa involvement**	12 (6.2)
**Histopathological characteristics (H&E)**	
Subepidermal blister	194 (100)
Inflammatory infiltrate	194 (100)
**Intensity of eosinophilic infiltrate (H&E)**	
Present, very intense	131 (67.5)
Present, not intense	59 (30.4)
Not present	4 (2)
**Direct immunofluorescence pattern (DIF)**	
Linear IgG + C3	131 (67.4)
Linear C3	47 (24.2)
Linear IgG	4 (2.1)
Negative DIF	8 (4.2)
Other	4 (2.1)

C3, C3 complement; H&E, Hematoxylin-Eosin; IgG, Immunoglobulin G; IgM, Immunoglobulin M; IgA, Immunoglobulin A; N, Total number; SD, Standard Deviation; %, Percentage.

Subepidermal blistering accompanied by the characteristic inflammatory infiltrate was present in all patients. In 67.5% the intensity of the eosinophilic infiltrate was very intense (>21 eosinophils/×40 high power field) (Fig. 1A). 93.7% presented linear deposits of IgG and/or C3 at the basal membrane in DIF. In 193 of them, collagen IV was detected on the floor of the blister (Fig. 1B).

**Figure 1:**
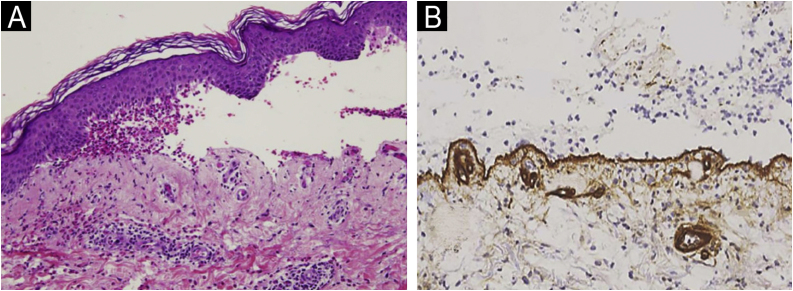
(A) Histopathological image of the blister from a BP patient (Hematoxylin & eosin, ×10): subepidermal blistering with an eosinophilic inflammatory infiltrate. (B) Collagen IV immunohistochemistry in BP (×20). Note the location of collagen IV (brown line) on the floor of the blister.

When comparing with EBA cases (confirmed by the presence of autoantibodies against collagen VII detected by ELISA [MESACUP anti-skin profile test]) attended in the same study period (n = 5), collagen IV was stained at the roof of the blister (Fig. 2). Salt-split skin DIF, probably due to technical limitations, lead to distinguish between BP and EBA in only 1 case.

**Figure 2:**
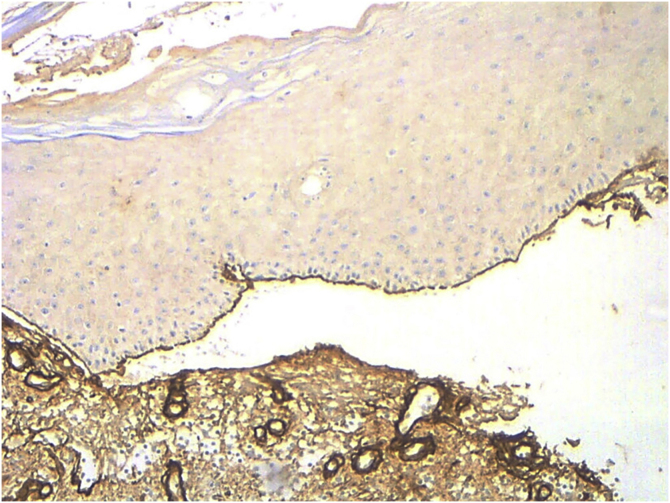
Collagen IV immunohistochemistry performed in an EBA patient (×10). Positivity is observed in the roof of the blister and in dermal structures that contain collagen IV, such as vessel walls.

In comparison with salt-split DIF, IHC is a simple and reproducible technique. It can be performed at the formalin-fixed paraffin-embedded tissue sections of the blister, without the need for additional processing.^6^, ^7^, ^8^ By demonstrating the location of collagen IV by IHC, the level of cleavage of blister may be established and, therefore, it can be helpful in the diagnosis of subepidermal ABD.^7^, ^8^

Collagen IV is located at the *lamina densa*. All the structures related to the hemidesmosome and *lamina lucida* [including BP Antigen 1 (BPAG1/BP230) and BP Antigen 2 (BPAG2/BP180), laminin 1 γ, laminin 332, α4β6 integrin] are located above *lamina densa*; collagen VII, however, is placed inferior to collagen IV.^8^ Therefore, when performing collagen IV IHQ in subepidermal ABD, except for EBA and bullous systemic lupus erythematous, the stain will be present on the floor of the blister (Table 2).^9^, ^10^

**Table 2:** Collagen IV immunohistochemistry in subepidermal autoimmune blistering diseases.

**Autoimmune subepidermal blistering disease**	**Antigen (pathogenic autoantibodies ‘target)**	**Collagen IV IHC** (performed in formalin-fixed skin biopsy of the bullae)
Bullous pemphigoid	BP180, BP230, p105, p200 (laminin 1γ)	Floor of the blister (dermal part)
EBA	Collagen VII	Roof of the blister (epidermal part)
Pemphigoid *gestationis*	BP180	Floor of the blister (dermal part)
Dermatitis *herpetiformis*	Tissue and epidermal transglutaminase (TG2, TG3)	Floor of the blister (dermal part)
Mucous membrane pemphigoid	BP180, BP230, laminin 332, α4β6 integrin	Floor of the blister (dermal part)
Linear IgA dermatitis	BP180, BP230	Floor of the blister (dermal part)
Bullous systemic lupus erythematous	Collagen VII	Roof of the blister (epidermal part)

BP, Bullous Pemphigoid; EBA, Epidermolysis Bullosa Acquisita; IHC, Immunohistochemistry; TG, Transglutaminase.

This technique has the limitation that only the location of collagen IV is being marked and the presence of immunoreactants or deposits cannot be evaluated. In the salt-split skin technique, the level of cleavage is obtained at the *lamina lucida* and, through DIF, the presence of immunodeposits can be assessed.

This study presents several limitations due to the lack of a control group, the scant number of EBA cases and the absence of other pemphigoid group and bullous disease patients.

Although widely known,^3^, ^4^, ^5^, ^7^, ^8^ after reviewing the published literature and through our observations, we consider that collagen IV IHC should gain visibility and presence in the diagnostic process of ABD.^9^, ^10^ We highlight this stain as a perfect first approach in the study of a blister, in general, and, especially, in the suspicion of ABD. Furthermore, gold standard techniques (DIF and/or salt-split skin DIF/IIF) results may be better interpreted along with collagen IV IHC findings.^3^, ^4^, ^5^, ^7^, ^8^, ^10^

## Financial support

None declared.

## Authors’ contributions

Lula María Nieto Benito: Conceived and designed the analysis, collected the data, contributed data or analysis tools, performed the analysis, wrote the paper and approved its final version.

Verónica Parra-Blanco: Conceptualization, Investigation, Validation and Writing, review and edit.

Ricardo María Suárez-Fernández: Conceived and designed the analysis, collected the data, contributed data or analysis tools, performed the analysis, wrote the paper and approved its final version.

## Conflicts of interest

None declared.
